# Front-face fluorescence of tetracyclines using a modulable 3D-printed platform modified with an extraction and sensing sorbent based on rare-earth metal–organic frameworks

**DOI:** 10.1007/s00604-025-07274-y

**Published:** 2025-06-08

**Authors:** Laura Alcázar-Escobedo, Noelia Campillo-Tamarit, Ernesto Francisco Simó-Alfonso, Enrique Javier Carrasco-Correa

**Affiliations:** CLECEM Group, Department of Analytical Chemistry, University of Valencia, 46100 Burjassot, Valencia Spain

**Keywords:** 3D printing, Metal–organic framework, Fluorescent sensor, Tetracyclines, Preconcentration

## Abstract

A stereolithographically modulable 3D-printed platform modified with a fluorescent metal–organic framework (MOF) has been developed for the selective recognition and extraction of tetracyclines, enabling their detection via front-face fluorescence spectroscopy. For this purpose, the MOF was grown in situ on the 3D-printed surface, which was previously functionalized with 2-aminoterephthalic acid, allowing the resulting composite to act as both sorptive and sensing material. Structural and morphological characterization of the MOF onto the 3D-printed device was performed using X-ray powder diffraction, scanning electron microscopy, infrared spectroscopy, and fluorescence analysis. The key advantage of the presented device is its full compatibility with conventional fluorometers, requiring no instrumental modifications since it fits directly into a standard cuvette holder. Additionally, the ability to perform measurements directly in the solid state, minimizing matrix effects thanks to the 3D-printed device functionalized with the MOF, offers a significant improvement over traditional methodologies. According to the Stern–Volmer model, the platform achieves good detection limits (1.8 and 0.5 μg L^−1^ in seawater and milk, respectively), inter-device variability below 12% and recoveries  exceeding 94%. Fabrication is cost-effective, with each complete unit costing only €1.60 including 3D printing, reagents, and labor (€0.5 per impact zone piece).

**Graphical Abstract:**

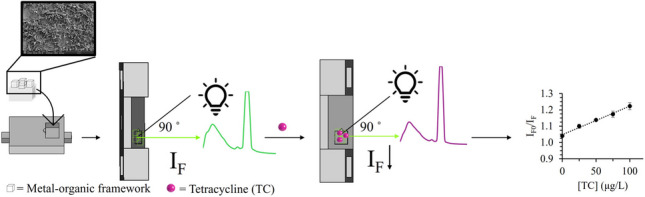

**Supplementary Information:**

The online version contains supplementary material available at 10.1007/s00604-025-07274-y.

## Introduction

3D printing is a technology offering innovative solutions across various disciplines such as aerospace [1], automotive [2], construction [3], self-healing materials [4], medical [5], and chemistry [6], among others [7–11]. The use of 3D printing offers significant advantages, including low-cost, rapid, and customizable fabrication of analytical devices. Unlike conventional manufacturing methods, such as photolithography or computer numerical control machining, it allows precise design control and easy integration of complex geometries, enabling the development of tailored platforms for specific applications. In chemistry, significant applications have emerged in Analytical Chemistry [6], including sample preparation [12, 13], separation science [14, 15], and sensing [16, 17].

3D printing operates by depositing material layer-by-layer to create tangible objects. Several methods have been developed over the years, with the most common being fused deposition modelling (FDM), stereolithography (SL) or vat polymerization, photopolymer inkjet printing (PIP), and selective laser sintering (SLS) [18]. Despite the advantageous features of PIP and SLS printing, their high costs have limited their widespread use across different fields. Conversely, FDM and SL have seen extensive application in Analytical Chemistry in recent years [6].

FDM involves the deposition of heated thermoplastic layer-by-layer, providing several advanced features such as the lack of need for post-processing, a wide variety of available materials, the ability to print multiple materials simultaneously, and low cost. On the other hand, SL employs a photopolymerizable resin and offers high-resolution prints through three submodes: (i) low-force SL (LFS), where a laser photopolymerizes the resin spot-by-spot and layer-by-layer; (ii) digital light processing (DLP), which uses a projector to emit light on specific pixels for complete layer polymerization; and (iii) masked SL (MSLA), which also uses a projector with a mask to selectively block light on non-polymerizing pixels. SL techniques require post-processing and offer limited material availability, typically printing only one material at a time. Therefore, SL provides smooth surface finishes, transparent/translucent materials, monolithic prints, easy machining, high resolution, and moderate-to-high solvent compatibility. These features make SL, particularly the LFS mode, highly valuable for Analytical Chemistry applications due to its high reproducibility and resolution.

In Analytical Chemistry, 3D printing has been used in many fields, but sample preparation and sensors have been the most explored. In the case of sample preparation, novel devices to improve different extraction modes have been developed [13], particularly those aimed at improving rotating disks for solid-phase extraction (SPE) [19–22]. Custom-designed and printed devices have addressed issues like the need for tedious evaporation and redissolution steps [20, 21] and 3D printing have provided novel ways to incorporate functional materials [19]. Regarding functional materials, metal–organic frameworks (MOFs) have been extensively used in sample preparation, particularly in SPE [23] but mainly in dispersive modes. Despite the simplicity of these modes, some disadvantages exist, making it interesting to explore their combination with other supports [24]. Nevertheless, its use in combination with 3D printing supports has been limited in sample preparation [25–27]. Combining MOFs with 3D printing has found more interest in catalysis [28] and remediation [29–32]. In any case, the combination of 3D printing with MOFs could be an interesting alternative to provide novel rotating disk systems in SPE.

3D printing has also been extensively used for sensor development [6], especially in electrochemical applications [16]. Optical sensors using 3D printing have also been studied, with fluorescence commonly used by incorporating fluorophores into gels for inkjet printing to detect sugars [33], DLP fluorophore-modified resin [34] or used as scaffolds [35]. Other approaches involve using 3D-printed devices modified with selective ligands and subsequent fluorophore modifications to construct sensors [22]. Additionally, MOFs can act as both extraction sorbents and fluorescent sensors [36–40]. In this context, lanthanide-based MOFs (Ln-MOFs) are particularly interesting as sensors due to their unique optical properties, such as high colour purity, visibly discernible colour, and relatively long luminescence lifetimes, originating from f-f transitions. The luminescence in MOFs occurs because the organic ligand absorbs energy and transfers it to the excited state of Ln^3+^, generating luminescence from the lanthanide ions [36]. Fluorescence quenching can also occur in the presence of compounds interacting with the MOF structure, which could be really interesting for analytical applications [40]. Hence, combining luminescent and extractant MOFs with 3D-printed devices can create specific, low-cost devices that enhance the performance of these materials.

Combining MOFs'dual roles as sensors and sorbents with 3D printing holds immense promise for advancing analytical applications, especially when paired with front-face fluorescence spectroscopy (F^3^S) techniques. F^3^S involves shallow-angle interaction of excitation and emission light with a sample's surface, reducing reabsorption and scattering, ideal for solid and opaque samples. The integration of 3D printing in F^3^S applications has been previously demonstrated [22], where a novel 3D-printed device, modified with plantibody, was used to detect microcystin-LR in seawater. This work highlighted the creation of multifunctional platforms enabling selective microscale extraction and direct surface sensing, eliminating unnecessary elution steps. Hence, combining F^3^S with MOF-based 3D-printed devices could thus yield highly sensitive and selective detection systems, leveraging the unique luminescent properties of MOFs and the non-limited creation characteristics of 3D printing.

In this context, several MOFs, particularly those based on Tb [40], have been shown to exhibit high selectivity for tetracycline detection. However, most of the reported methods rely on dispersive sensing formats and often fail to achieve detection limits (LOD) low enough for application in challenging matrices such as seawater [41, 42] or milk [43]. Therefore, the integration of MOFs with a 3D-printed platform to enhance analytical performance represents a promising advancement in the field of MOF-based sensing. This work aims to develop an advanced, modular 3D-printed platform for F^3^S integrated with luminescent Ln-MOFs that are also able to act as SPE sorbent. To our knowledge, integrating MOFs with 3D printing for both extraction and sensing applications has not yet been explored. This study enhances a previously designed 3D-printed system [22] by leveraging LFS to refine its capabilities. The open-source F^3^S solid devices will mimic the dimensions of cuvettes used in conventional fluorescence spectrometers, ensuring compatibility with commercial equipment. Additionally, new 3D-printed components are designed to optimize MOF incorporation and improve the rotative SPE. The efficacy of the extraction device is demonstrated through the determination of tetracyclines in challenging samples, such as seawater and milk.

## Materials and methods

### Reagents

During the execution of the work, the following reagents were used: hydrochloric acid (HCl), dimethyl sulfoxide (DMSO), ethanol (EtOH), sodium hydroxide (NaOH), isopropanol (IPA), and malic acid (MA) supplied by VWR (Rosny-sous-Bois, France). Magnesium chloride hexahydrate and a 32% ammonia solution were obtained from EPICA S.L. (Barcelona, Spain). On the other hand, acetonitrile (ACN), formic acid (FA), and methanol (MeOH) were obtained from Labbox (Barcelona, Spain). N-Hydroxysuccinimide (NHS) was provided by Sigma-Aldrich (Darmstadt, Germany), while terbium(III) nitrate hexahydrate, 2-aminoterephthalic acid (ATPA), and (1-ethyl-3-(3-dimethylaminopropyl)carbodiimide hydrochloride (EDC) were acquired from Fisher Scientific (Madrid, Spain). Lastly, trimesic acid (BTC) used in this work was supplied by Tokyo Chemical Industry (Tokyo, Japan).

The tetracyclines (tetracycline, TC, oxytetracycline, OTC, and chlorotetracycline, CTC) used for the analyses were provided by Alfa Aesar (Massachusetts, USA). Other analytes employed to study selectivity were ciprofloxacin (CPF) from Alfa Aesar, ibuprofen (IBU) from Guinama (Valencia, Spain), and p-dinitrophenol (pDNP) and bisphenol A (BPA) from Sigma-Aldrich. The 3D printing parts were fabricated using Clear IV resin (RS-F2-GPCL-04) supplied by FormLabs (Somerville, MA, USA). Nanopure water was obtained using a Crystal B30 Adrona deionizer (Riga, Latvia). Synthetic seawater was prepared following the recommendations by Wetzel and Likens [43].

### Intruments

3D devices were printed using a Formlabs Forms 3 LFS 3D printer and later cured using a UV chamber model CL 1000 from UVP Inc. (Upland, CA, USA). The modification of the 3D device was performed using a Memmert oven (Schwabach, Germany). Characterization of the MOF and 3D-printed parts was done using a Fourier-transform infrared spectroscopy system FTIR-ATR 4100 (Jasco, Madrid, Spain), a scanning electron microscope (SEM) model SCIOS 2 FIB-SEM (ThermoFisher Scientific, MA, USA), and an X-ray powder diffraction (XRD) system model D8 Advance A25 (Bruker, MA, USA). The extraction capacity of tetracyclines by the hybrid 3D printing-MOF system was assessed using HPLC with UV detection on a 1200 system (Agilent, CA, USA). Fluorescence measurements of the MOF and the 3D-printed pieces, with and without MOF, were performed using a MOD FP-6200 spectrofluorometer (Jasco).

Chromatographic separations were carried out using a Jasco HPLC system (model 2000) equipped with a fluorescence detector, while mass spectrometry measurements were conducted using an Agilent liquid chromatograph coupled to a triple time-of-flight mass spectrometer (TripleTOF) from SCIEX (TRIPLETOFT6600plus). For seawater samples in mass spectrometry, desalination was necessary to minimize matrix effects, as described later. A Hidrowater desalination system (Aldaya, Valencia) model Ro-0206–12 was used, which reduced conductivity by a factor of 50. The activated carbon filter was removed from this system to minimize the risk of tetracycline retention in this type of material.

### Tb-based MOF synthesis for dispersive mode

To synthesize a fluorescent (Tb(BTC)(H_2_O)_6_) MOF, the procedure described by Bhardwaj et al*.* [45] was used. For the synthesis, 10 mL of a 0.01 M aqueous solution of Tb(NO_3_)_3_·6H_2_O was mixed with 10 mL of a 0.01 M ethanolic solution of trimesic acid. The mixture was then stirred for 3 h at room temperature. After this time, the dispersion containing the MOF was centrifuged at 4500 rpm for 5 min, and the supernatant was discarded. The solid was washed with ethanol, and the centrifugation procedure was repeated. The process was repeated with water, and then the still-wet solid was placed in an oven at 60 °C for at least 24 h.

### Design and printing of the 3D-printed devices

**Fig. 1:**
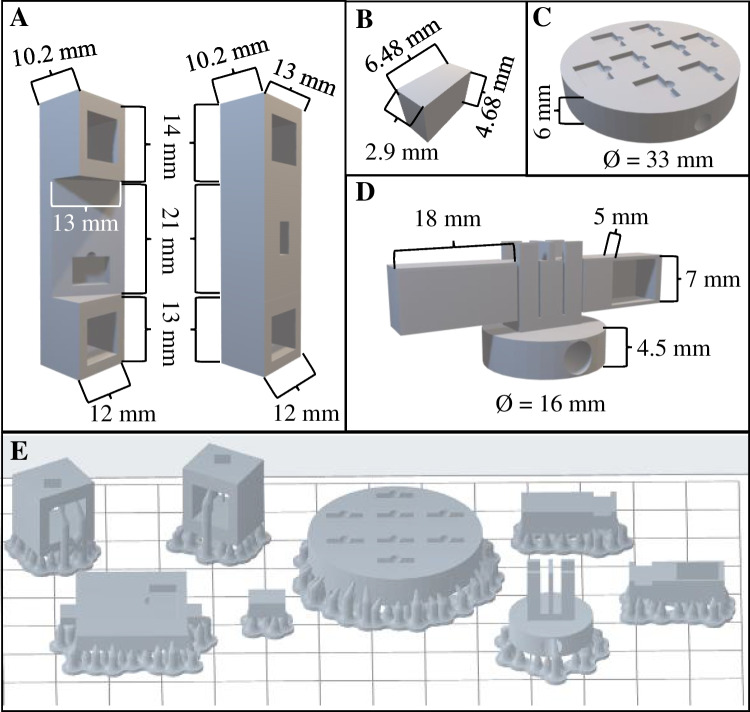
3D-printed designs of the F^3^S cell (A), impact zone (B), reaction support (C) and the extraction device (D) and the orientation and supports needed to print all the parts (E)

Four components were designed using FreeCAD (an open-source CAD software) [46] for this work. First, a support was designed to be used as a F^3^S cell based on previous studies (Fig. 1A) [22]. Briefly, the cell is composed of three parts: a central section with space for a magnet and a frontal groove measuring 7.2 × 3.2 × 5.2 mm in length, width, and height, respectively, two lateral supports to fit properly into the cuvette slot of the fluorescence benchtop instrument. The shapes and dimensions are shown in Fig. 1A. Figure 1B shows a rectangular prism, referred to as the impact zone, which aligns with the groove in the central section of the cell (Fig. 1A) and corresponds to the impact area of the excitation beam in the fluorescence instrument. This piece was modified and used for the fluorescent MOF growth. The third component, the reaction cylinder (Fig. 1C), was designed to facilitate the synthesis of the MOF on the impact zone piece. Finally, the fourth component designed was used for stirring along the extraction process (Fig. 1D). It consists of two parts: an agitation area and two removable arms, one of which can hold the “impact zone” piece during the extraction process. The STL files can be found in the supplemental material.

To print the components, the aforementioned designs were imported into the printer software for proper orientation and to add the necessary supports for printing (Fig. 1E). In general, the working surface is positioned opposite to the printing platform to avoid surface defects. Subsequently, a washing and curing process was performed to remove any remaining resin from the components and complete the polymerization. For this purpose, the printed components were immersed in IPA and placed in an ultrasonic bath for 15 min. This process was repeated once with nanopure water and again with IPA. The components were then dried with paper and N_2_ to remove any residual IPA from the surface and placed in a UV chamber with 254 nm lamps for 1 h at 1 J cm^–2^. Once cleaning and curing were completed, the components were stored until use.

### Surface modification and Tb(BTC)(H_2_O)_6_MOF growth of the 3D-printed impact zone

To anchor the Tb(BTC)(H_2_O)_6_ MOF onto the surface of the 3D-printed impact zone (Fig. 1B), a sequence of reactions to generate the appropriate functional groups was performed, based on a procedure previously described [47]. Subsequently, the Tb(BTC)(H_2_O)_6_ MOF was generated through direct growth based on the work developed by Gan et al*.* [48]. First, the front-face of the impact zone, was placed on 55 μL of a 2 M NaOH aqueous solution in a Petri dish and reacted in an oven at 60 °C for 30 min. After this period, it was cleaned with a few drops of 0.1 M HCl and water and then allowed to dry at room temperature. Next, the front-face of the piece was again placed on 55 μL of an EDC (0.2 M) and NHS (0.3 M) solution in water and incubated in the oven at 60 °C for 30 min, followed by a water wash. Subsequently, the front-face of the piece was contacted with 55 μL of a 0.5 M aminoterephthalic acid solution in DMSO and incubated in the oven at 60 °C for 60 min, followed by washes with MeOH and water, and dried at room temperature for 24 h.

For the MOF growth, a one-step growth procedure on surfaces with carboxylic groups was chosen [49]. For this purpose, the previously modified impact zone piece was placed in the reaction cylinder (Fig. 1C), which was then inserted into a beaker for MOF synthesis following the procedure described in Synthesis of the MOF for dispersive mode Section. Briefly, solutions of 0.01 M Tb(NO_3_)_3_·6H_2_O in water and 0.01 M BTC in EtOH were mixed, and stirring was achieved with a magnet placed at the base of the reaction cylinder. The reaction process was maintained for 6 h, followed by cleaning with a 1:1 EtOH:H_2_O mixture, a 1:1 ACN:DMSO (1% formic acid) mixture, and water. Finally, the piece was allowed to dry at room temperature until ready for use in the determination of tetracyclines by front-face fluorescence.

### Analysis of tetracyclines using dispersive methodology

To determine the concentration of tetracyclines in water using their quenching effect on the MOF in dispersive mode, the following procedure was followed: In a 10 mL volumetric flask, 0.5 mL of a 100 mg/L dispersion of the Tb(BTC)(H_2_O)_6_ MOF in water, previously sonicated, was added. Then, the appropriate volume of the standard or sample containing TC was added, and the flask was filled to the final volume with water. The dispersion was then placed on a vortex mixer for 1 min, and the fluorescent signal was measured in a fluorescence cuvette using an excitation wavelength of 270 nm. Emission was measured at 541 nm, with a correction applied at 515 nm. The correction involved subtracting the fluorescence intensity (I_F_) at 515 nm from the I_F_ at 541 nm. Between each measurement, the cuvette was cleaned with 1 M HCl and water to remove any residual remaining Tb-based MOF.

### Analysis of tetracyclines using 3D-printed devices

**Fig. 2:**
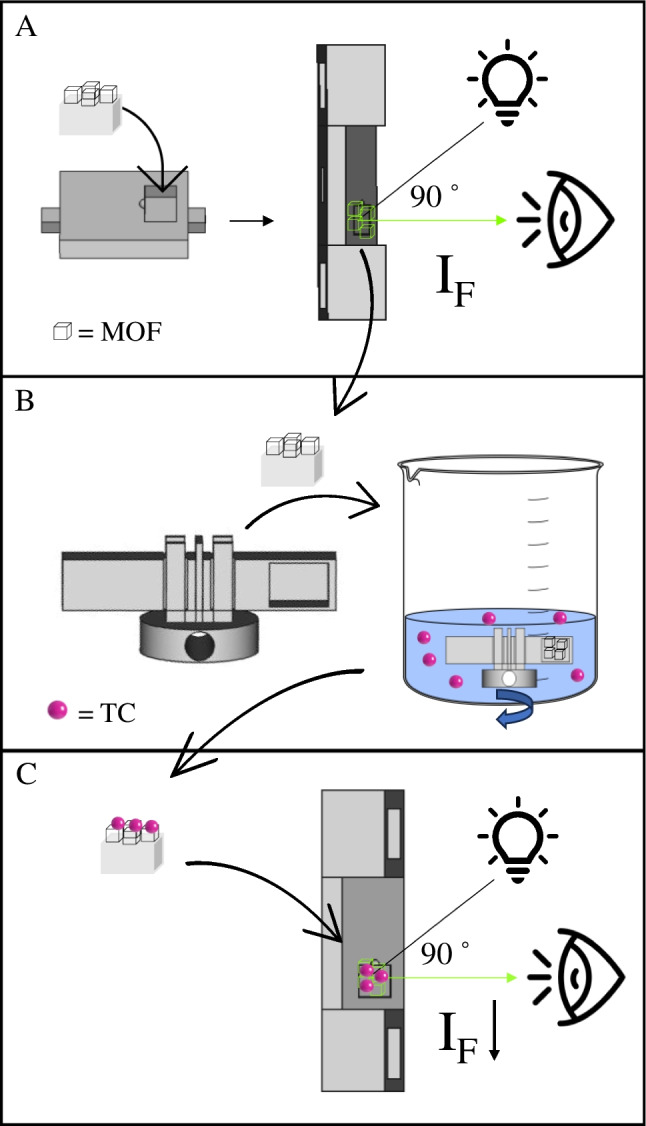
Scheme of the fluorescence measurement procedure on 3D-printed devices containing Tb(BTC)(H_2_O)_6_ MOF. The figure illustrates the measurement taken before extraction (**A**), the extraction process (**B**), and the measurement taken after the extraction of TC (C)

The protocol for using the 3D pieces containing the Tb(BTC)(H_2_O)_6_ MOF as tetracycline sensors is outlined in Fig. 2. For this purpose, the impact zone 3D-printed pieces modified with the Tb(BTC)(H_2_O)_6_ MOF (see Surface modification Section) were placed in the front-face groove of the F^3^S cell (Fig. 2A), excited at 294 nm, and their emission spectrum was recorded for later signal correction. Next, the same impact zone pieces were secured onto the arm of the extraction device (Fig. 1D) with a hollow cavity using adhesive carbon tape and placed in the stirring platform. A counterbalance arm was placed on the opposite side to offset the weight (Fig. 2B). The extraction system containing the impact zone pieces was stirred using a magnet positioned at the bottom of the stirring platform in the presence of 25–100 mL of tetracycline standard or sample without dilution for 30 min. After this time, the impact zone piece was returned to the F^3^S cell (Fig. 2C) and fluorescence was measured by exciting at 294 nm, recording the spectrum between 350 and 700 nm.

### Chromatographic methodologies

To evaluate the retention of TC on the Tb(BTC)(H_2_O)_6_ MOF grown on the surface of the impact zone piece and/or to compare it with other conventional methodologies for TC detection, a chromatographic method was performed using a Kinetex XB-C18 and a linear gradient from 95:5 H_2_O (0.1% formic acid):ACN to 100% ACN over 6 min. For UV detection, the wavelength was set to 280 nm, with a flow rate of 1 mL/min and an injection volume of 20 μL. For fluorescence detection, the excitation wavelength was 375 nm, and emission was measured at 535 nm, with a flow rate of 1 mL/min and an injection volume of 20 μL. For MS, the flow was set at 0.4 mL/min and the injection volume was 5 μL. Measurements in MS were using TripleTOF at a mass range of 445.1505–445.1705 in SIM mode. The MS parameters were set to positive mode, ion source gas 1 at 60 psi, ion source gas 2 at 60 psi, curtain gas 1 at 30 psi, temperature at 550 °C and ion spray voltage at 5500 V. The accumulation time was set at 240 ms.

## Results and discussion

### Preliminary studies

First, the synthesis of the Tb(BTC)(H_2_O)_6_ MOF in powder was carried out using a stirring-assisted precipitation method, at different reaction times, with green solvents (water and ethanol) at room temperature [45, 48]. The resulting MOF was characterized using XRD and SEM (Fig. S1) and fluorescence spectroscopy (Fig. S2) showing the typical patterns found in literature [45, 48], indicating successful synthesis of the desired MOF. The fluorescence emission spectrum (λ_exc_ = 260 nm) of the dispersed in water Tb(BTC)(H_2_O)_6_ MOF is shown in Fig. S2 A. The maximum excitation wavelength was determined from the excitation spectrum at 541 nm. The emission spectrum showed the characteristic transitions of the Tb^3+^ at 489, 541, 584, and 617 nm, corresponding to the ^5^D_4_-^7^F_6_, ^5^D_4_-^7^F_5_, ^5^D_4_-^7^F_4_, and ^5^D_4_-^7^F_3_ transitions, respectively [45, 50].

The fluorescence intensity (Fig. S2B) at different reaction times measured at 541 nm, the quenching effect (Fig. S2 C) of a 5 mg L^–1^ TC solution prepared in the presence of the Tb(BTC)(H_2_O)_6_ MOF (5 mg L^–1^) and the Tb(BTC)(H_2_O)_6_ MOF concentration (Fig. S2D) were used to determine the optimal reaction time. The highest quenching efficiency, 64 ± 2%, was observed for the MOF synthetized in 3 h and 5 mg/L. The results, as shown in Figs. S2E and S2 F, demonstrated strong linearity within this range, following the Stern–Volmer equation [51] (Eq. 1):

1 $$\frac{{I}_{F0}}{{I}_{F}}=1+{K}_{SV}\cdot \left[TC\right].$$

where ${I}_{F0}$ represents the corrected intensity of the Tb(BTC)(H_2_O)_6_ MOF at 541 nm, and ${I}_{F}$ is the corrected fluorescence intensity in the presence of TC, obtained from calibration at various concentrations. Here, *K*_*SV*_ is the quenching constant, and *[TC]* is the concentration of TC. The calibration curve exhibited an R^2^ correlation of 0.975 ($\frac{{I}_{F0}}{{I}_{F}}=1.11+0.25\cdot [TC]$). The *K*_*SV*_ reflects the sensitivity of the calibration curve, with the synthetized Tb(BTC)(H_2_O)_6_ MOF in this study displaying a value of 0.25 L mg^–1^ or 110 L mol^–1^. This sensitivity is up to 38 times larger than those previously published in literature for other La-based MOFs [48]. Additionally, the sensitivity was further evaluated using other TCs (OTC and CTC) and emerging pollutants, such as CPF, pDNP, BPA, and IBU. All tested TCs showed similar quenching percentages (56–64%), whereas other compounds, including CPF, pDNP, and BPA, exhibited quenching effects below 10%. The analytical performance of the method in water was evaluated and compared to the corresponding parameters of HPLC-FLD and HPLC–MS methods (Table S1). The limit of detection (LOD) for the developed dispersed fluorescence method, was determined to be 140 μg L^−1^. This value is higher than those obtained with HPLC-FLD (0.3 μg L^−1^) and HPLC–MS (7 ng L^–1^) methods. On the other side, the relative standard deviation (RSD) and linearity were comparable across the three methods. The sensitivity achieved by the fluorescence MOF (0.250 L mg^–1^) represents an intriguing intermediate value. It outperforms HPLC-FLD (0.099 L mg^–1^) but remains below the sensitivity of HPLC–MS (1.009 L mg^–1^). Additionally, the Tb(BTC)(H_2_O)_6_ MOF was also tested to measure the TC in seawater and the results showed that the effect of the ionic strength produces a quenching effect. For this reason, a seawater sample was desalted (see Instrumentation Section) but still strong matrix effect (70% slope reduction) was observed. Overall, while the developed method demonstrates inferior analytical performance relative to conventional methods, its advantages include lower operational costs and a greener approach to analysis. In any case, to address the limitations of this proposed methodology, incorporating advanced techniques such as 3D printing and F^3^S into the methodology is proposed as will be developed in the following sections. These innovations have the potential to enhance the analytical performance, making the method more competitive while maintaining its cost-effectiveness and environmental benefits.

### Design, synthesis and characterization of the 3D-printed F^3^S devices containing the Tb(BTC)(H_2_O)_6_ MOF

**Fig. 3:**
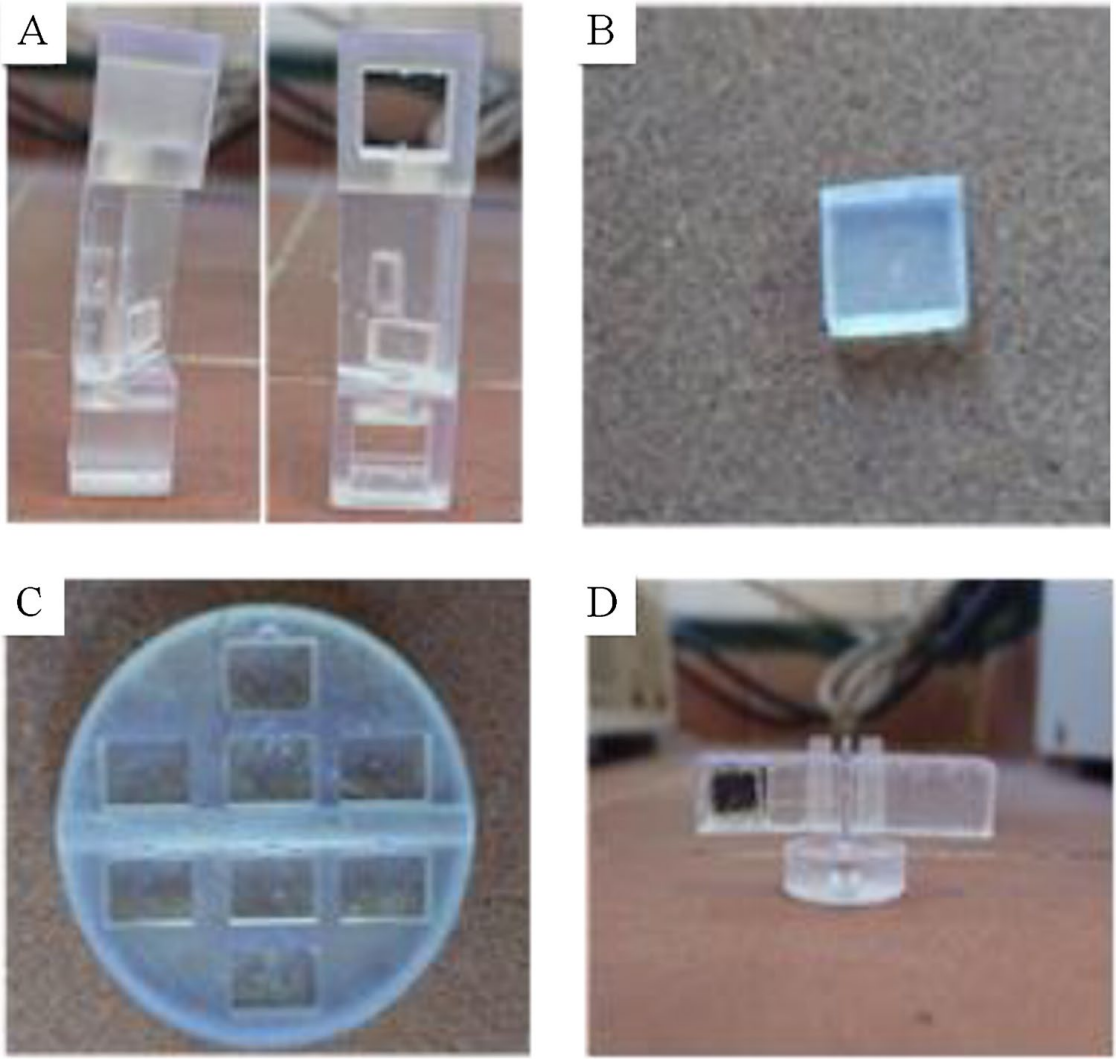
3D-printed F^3^S cell (**A**), impact zone (**B**), reaction support (**C**) and the extraction device (**D**)

In this study, four distinct devices were designed and fabricated using the SL 3D printing methodology to facilitate different stages of the extraction and sensing process (see Figs. 1 and Fig. 3). These devices include the F^3^S cell (Figs. 1A, 3A), the impact zone (Figs. 1B, 3B), the reaction support (Figs. 1C, 3C), and the extraction device (Figs. 1D, 3D). The F^3^S cell (Figs. 1A, 3A) was adapted from a previously developed design [22], with improvements aimed at reducing the amount of sorbent required. This was achieved by introducing an impact zone device (Figs. 1B, 3B). In the original design, the F^3^S cell consisted of three zones: two lateral supports and a central part. The surface of the central part was entirely modified with an antibody to perform both the extraction and sensing processes. However, the fluorescence instrument's excitation beam only interacts with a small area located on the lower section of the central device. To address this inefficiency, we decided to design and print the impact zone device. This simple cuboid has an area matching that illuminated by the fluorescence excitation beam and is designed to fit precisely within a specially designed slot in the F^3^S cell. The slot includes a small indentation to facilitate the easy exchange of impact zone devices within the same F^3^S cell, thereby enhancing the flexibility and efficiency of the system. In addition to the two previously described devices, two additional devices were designed and 3D-printed to optimize the surface synthesis of the Tb(BTC)(H_2_O)_6_ MOF on the impact zone, the reaction support (Figs. 1C, 3C), and to improve the extraction process, the extraction device (Figs. 1D, 3D). The reaction support enables the simultaneous synthesis of up to eight impact zone devices, significantly reducing both processing effort and time. This is achieved by fitting eight impact zone devices into dedicated slots with their selected front faces positioned upward. Additionally, the reaction support includes a cylindrical hole designed to accommodate a magnet, which facilitates stirring along the synthesis process. The extraction device is a specialized stirring tool that features a central section with a cylindrical hole for a magnet and two removable arms. One arm is solid, while the other includes a slot designed to securely hold the impact zone device, with the help of carbon tape, during the extraction process. This configuration ensures efficient contact of the front face of the impact zone in the extraction process.

The printing and cleaning processes were conducted as detailed in Design and printing of the 3D-printed devices Section. Briefly, the printing process was designed to ensure that the front face of the impact zone device was oriented away from the printing platform. This orientation was chosen to achieve a smooth surface, free from surface roughness caused by resin accumulation or the presence of supports. The other devices were printed as illustrated in Fig. 1E. The cleaning process followed the methods described in previous works [22, 52, 53] to eliminate resin residues or sticky parts that could interfere with fluorescence measurements.

**Fig. 4:**
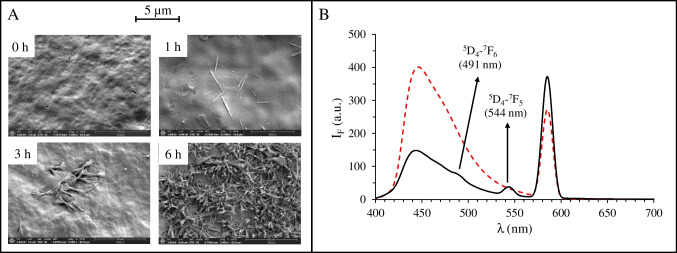
SEM micrographs of the 3D-printed surface modified with NaOH and APTA (0 h) and further modified with Tb(BTC)(H_2_O)_6_ MOF after 1, 3, and 6 h of growth (**A**). All micrographs were captured at 5,000 × magnification, except for the 1-h sample, which was taken at 25,000 × for enhanced structural detail. Emission spectra (λ_*exc*_ = 294 nm) of the 3D-printed device modified with NaOH and APTA (red dashed line) and after modification with Tb(BTC)(H_2_O)_6_ MOF for 6 h of growth (black line) (**B**)

To immobilize the Tb(BTC)(H_2_O)_6_ MOF onto the surface of the impact zone device, a direct growth procedure was followed (see Surface modification Section). Briefly, the ester 3D-printed surface of the methacrylate impact-zone devices was modified with NaOH to hydrolyze the surface into carboxylic moieties. This was followed by the covalent attachment of ATPA through the amine group using the carbodiimide protocol (EDC-NHS) in two steps. The success of these reactions was monitored by FTIR (Fig. S3 A). The C = O band of the ester in the 3D-printed resin (1701 cm^−1^) showed a shoulder after modification with NaOH (1694 cm^−1^) due to the presence of carboxylic moieties. Another shoulder appeared after the EDC-NHS and ATPA reactions (1718 cm^−1^) due to the formation of an amide bond. Additionally, bands at 950, 1451, 2100, and 3384 cm^−1^, corresponding to N–H flexion, C-N bend, aromatic ring, and N–H bend, respectively, appeared after modification with ATPA. These findings demonstrate the successful modification of the surface. This modified surface acts as an ideal base for the direct growth [49] of the Tb(BTC)(H_2_O)_6_ MOF. It facilitates the creation of the initial layer between the attached APTA and the metal (Tb). Subsequently, the reticular structure will grow over time on the surface. As observed in Fig. S3B, the presence of the Tb(BTC)(H_2_O)_6_ MOF is confirmed by the most intense peaks of its XRD pattern (Fig. S1), which also appear in the XRD pattern of the 3D-printed surface modified with the MOF at 6 h reaction time at 13.4°, 17.9°, 18.4°, and 19.3°. Additionally, the growth of the Tb(BTC)(H_2_O)_6_ MOF on the APTA-modified 3D-printed surface was studied morphologically using SEM (Fig. 4A) and energy-dispersive X-ray spectrometry (EDX) over a time range of 1 to 6 h. As observed in the micrographs, the presence of the Tb(BTC)(H_2_O)_6_ MOF was minimal at 1 and 3 h but increased significantly after 6 h. This trend was further confirmed by EDX analysis, which showed that the Tb surface percentage remained below 0.1% at 1 and 3 h, rising to approximately 0.6% after 6 h of growth.

Regarding fluorescence, Fig. 4B presents the emission spectra obtained at an excitation wavelength of 294 nm for the APTA-modified 3D-printed surface (red dashed-line) and the surface modified with the Tb(BTC)(H_2_O)_6_ MOF after 6 h of growth (black line). The emission spectrum of the APTA-modified 3D-printed surface exhibits two prominent bands at approximately 450 and 585 nm. After modification with the MOF, the intensity of the 450 nm band decreased by more than 2.5 times, whereas the 585 nm band increased by approximately 1.3 times. The reduction in the 450 nm band intensity could be attributed to reduced accessibility of the excitation beam to the 3D-printed surface due to MOF coverage, while the enhancement of the 585 nm band may result from a synergistic effect between the MOF’s characteristic emission at 584 nm (corresponding to the ^5^D_4_-^7^F_4_ transition) and the fluorescence of the 3D-printed surface. Additionally, the two most intense characteristic transitions of the MOF, ^5^D_4_-^7^F_6_ and ^5^D_4_-^7^F_5_, were observed at 491 and 544 nm, respectively, in the MOF-modified 3D-printed surface. The emission band at 544 nm (with quotient correction at 515 nm) was selected for further studies as it exhibited the highest intensity and minimal spectral overlap.

### Tetracycline sensing using the 3D-printed F^3^S device modified with the Tb-based MOF

Fluorescence intensity and quenching effects at 544 nm (corrected at 515 nm) were also evaluated for different MOF growth times (1, 3, and 6 h). The results indicated that the 6-h growth time yielded the highest fluorescence intensity with the better reproducibility (1.30 ± 0.05 a.u.), compared to 1 h (1.0 ± 0.5 a.u.) and 3 h (0.8 ± 0.2 a.u.). To evaluate the usability of the 3D-printed devices modified with the Tb(BTC)(H_2_O)_6_ MOF, their extraction capability of the 6-h MOF growth was assessed using HPLC. This evaluation is essential since the measurements will be performed on the dry surface of the impact zone (Fig. 1B and 3B) rather than in solution. Thus, it is crucial that TC is either quantitatively retained by the MOF or establishes an equilibrium that allows the measurements to correlate with its concentration. For this purpose, TC solutions with concentrations ranging from 0.05 to 1.5 mg L^−1^ were brought into contact for 30 min with the impact zone containing the Tb(BTC)(H_2_O)_6_ MOF placed on the center part of the F^3^S device (Fig. 1A and 3A). As described in a previous study [22], the device includes a designated position for a magnet, enabling the F^3^S cell to stir the solution containing TC. However, HPLC analysis revealed that no significant retention of TC occurred on either the surface of the impact zone or the 3D-printed structure. To address this issue, an alternative extraction setup (Fig. 1D and 3D) incorporating a carbon tape was implemented. This modification allowed the impact zone to be stirred more effectively, enhancing the contact between the MOF-modified surface and the surrounding sample or TC solution. Repeating the extraction study under these improved conditions demonstrated that, regardless of the initial concentration, 48 ± 5% of TC was consistently retained, confirming the establishment of an extraction equilibrium between TC and the surface-grown MOF. The extraction setup without the impact zone modified with the MOF did not show TC retention. Extended contact times were also tested, but the results remained unchanged. In any case, the modified 3D-printed device is suitable for use as a TC sensor. The quenching effect was evaluated for all three tested growth durations, with the most pronounced effect observed at 6 h. This condition also exhibited the highest reproducibility (33 ± 1%) compared to 1 h (10 ± 4%) and 3 h (5 ± 4%). Based on these results, the 6-h growth time was selected as the optimal condition for further experiments.

**Fig. 5:**
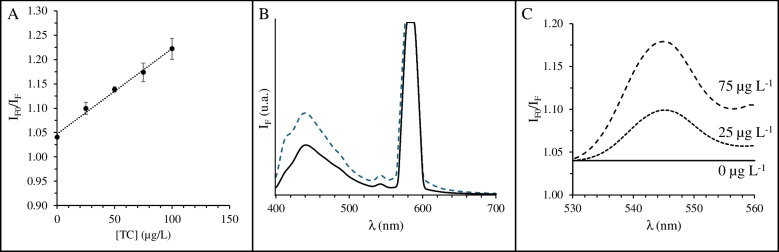
Calibration curve fitted using the corrections and the Stern–Volmer equation (**A**), emission spectra of the 3D-printed device modified with Tb(BTC)(H_2_O)_6_ MOF before (blue dashed line) and after (black line) the TC extraction at 75 μg L^–1^ (**B**), and Stern–Volmer corrected spectra obtained by using the applied correction to the obtained spectra after the extraction at 0, 25 and 75 μg L^–1^ (**C**). All the fluorescence emission intensity was obtained with an excitation wavelength of 294 nm

As in Preliminary studies Section, the linearity of the quenching effect for TC was assessed by constructing a calibration curve over a concentration range of 25 to 100 μg L^−1^ under optimal conditions. The results (Fig. 5A) confirmed that the system follows the Stern–Volmer equation (Eq. 1). The calibration curve exhibited a strong linear correlation (R^2^ = 0.9899), with the equation $\frac{{I}_{F0}}{{I}_{F}}=1.05+0.0017\cdot [TC]$, indicating that the fluorescence quenching effect aligns with the Stern–Volmer model. This behavior is consistent with a static (complexation) quenching process, as can be inferred by the HPLC extraction experiments and the sensing procedure. In previous studies in dispersive mode using Tb-based MOFs [54, 55], a mixed quenching mechanism, both static and dynamic, was observed. However, in the present study, fluorescence measurements are carried out in the solid state, where molecular diffusion is limited. Under these conditions, the quenching is most likely dominated by static interactions. This suggests that TC may be adsorbed or coordinated to the MOF surface, supporting a static quenching model. The *K*_*SV*_ was determined to be 1.7 L mg^−1^ (756 L mol^−1^) for the synthesized Tb(BTC)(H_2_O)_6_ MOF onto the 3D printed surface of the impact zone. This sensitivity is up to 7 times higher than that obtained for the dispersive mode explored in Preliminary studies Section, and up to 260 times greater than values previously reported for other La-based MOFs [48]. As an example, Fig. 5B presents the emission spectra of the 3D-printed device before (blue dashed line) and after (black line) extraction, highlighting the fluorescence quenching effect induced by TC adsorption. Additionally, Fig. 5C shows the Stern–Volmer spectra at TC concentrations at 0, 25 and 75 μg L^–1^, obtained by applying the aforementioned corrections to the emission spectrum of the 3D-printed device after the extraction. As shown, the increasing quenching effect is evidenced by the progressive enhancement of the Stern–Volmer signals corresponding to the ^5^D_4_-^7^F_5_ transition band.

The breakthrough volume of the 3D-printed device modified with Tb(BTC)(H_2_O)_6_ MOF (Fig. S4) was evaluated over a volume range of 25–250 mL using 1.25 μg of TC. The recovery results indicated that the system maintained a high retention efficiency, with only a minor decrease of 12% when the volume increased from 25 to 100 mL. However, a sharp decline to 29% recovery was observed when the loading volume was further increased to 250 mL. The prepared 3D-printed devices were stable for at least one month without detectable loss in performance. The LOD for the developed 3D-printed fluorescence method, using the Tb(BTC)(H_2_O)_6_ MOF for TC quantification, was determined to be 1.6 μg L^−1^. This value is two orders of magnitude lower than that obtained with the dispersed strategy described in Preliminary studies Section, underscoring the superior sensitivity of the surface-grown MOF approach. Moreover, this LOD is comparable to that achieved using direct HPLC-FLD methodology with simple instrumentation. Additionally, the RSD for a 50 μg L^−1^ TC measurement across different 3D-printed devices was 11.2%, which is also lower than that observed for the dispersed mode, confirming the improved reproducibility of the proposed method. However, on the 3D-printed device, the increase in ionic strength leads to a small decrease of the fluorescence signal after extraction. Despite this reduced effect, the quenching induced by TC remains observable and continues to follow the Stern–Volmer equation in synthetic seawater, described as: $\frac{{I}_{F0}}{{I}_{F}}=2.03+0.0015\cdot [TC]$, where the *K*_*SV*_ is 1.5 L mg^–1^. To account for the matrix effect, a matrix-matched calibration curve was established using synthetic seawater to ensure accurate quantification. The LOD in seawater was 1.8 μg L^−1^. The proposed methodology was applied to a real seawater sample collected from the port of Valencia (39° 27′ 42″ N, 0° 18′ 34″ W). The sample showed TC concentrations below the LOD. To evaluate the matrix effect in seawater, two spiking levels (10 and 75 μg L^−1^) were selected, as no official maximum residue limits (MRLs) are established for TCs. These concentrations were chosen based on the platform’s linear response range and their relevance for environmental monitoring purposes [41, 42]. The results showed recoveries of 99.6 ± 1.3% and 96 ± 4%, respectively. Additionally, the proposed method, using standard addition methodology, was applied to the analysis of spiked bovine milk samples using the same concentrations (under the MLR in milk [43]) as those tested in seawater. The resulting Stern–Volmer constant (5.8 L mg^–1^) was even higher than that obtained in standard solutions and seawater, indicating the presence of a matrix effect. However, this effect did not compromise the method’s sensitivity, as the LOD in milk was even lower than that in seawater, reaching 0.5 μg L^–1^. Furthermore, recovery rates remained satisfactory, with values of 94 ± 7% and 99 ± 7% for concentrations of 10 and 75 μg L^–1^, respectively.

Furthermore, the proposed method achieves LODs that are comparable to or lower than those reported in recent literature (Table 1). The use of the 3D-printed F^3^ device significantly enhances the system's performance compared to conventional MOF-based approaches, particularly those employing MOFs in dispersive mode [56]. In the analysis of TC in seawater, the F^3^ device provided a LOD of 1.8 μg L^–1^, which is superior to many electrochemical detection systems (e.g., 4.5 μg L^–1^ in environmental water [57]). However, it still falls short of the ultra-trace LODs obtained by high-end methods such as HPLC–MS (see Preliminary studies Section). In the case of milk analysis, the proposed method also demonstrates strong performance, with an LOD of 0.5 μg L^–1^. This is comparable to, and in some cases better than, traditional methods such as HPLC–DAD after SPE (21 μg L^–1^) [58] or electrochemical sensors using standard materials (0.2 μg L^–1^) [59]. Overall, the F^3^ 3D-printed device offers a practical alternative to more complex and time-consuming analytical techniques.

**Table 1:** Comparison of the proposed method with previously reported analytical approaches for the detection of tetracyclines in various matrices

Sample preparation	Anaysis	Sample	LOD (μg L^–1^)	RSD (%)	Recovery (%)	Reference
Ultrasonic assisted extraction	Ratiometric fluorescence	Fish feed Pork samples	88	6.7	93–102 94–110	[56]
QuEChERS + lyophilization	HPLC-FLD	Chicken breast	30	5.0	80–101	[60]
SPE – Oasis HLB cartridges	HPLC–DAD	Milk	21	9.2	90.2–108.1	[58]
SPE—Ionic liquid	HPLC–MS/MS	Milk	0.28	4.8	99.4–100	[61]
-	Electrochemical	Waters	4.5	4.8	95–97	[57]
-	Electrochemical	Standards	49	4.5	-	[62]
-	Electrochemical	Milk	0.2	6.8	98–104	[59]
-	-SPE-F^3^S	Seawater Milk	1.8 0.5	11.2	96–99.6 94–99	This work

## Conclusions

In this study, a low-cost analytical platform was developed by integrating a terbium-based MOF onto a SL 3D-printed structure for the simultaneous extraction and detection of TCs via F^3^S. This hybrid device represents a significant improvement over traditional dispersive sensing systems. While dispersive methods are commonly used, they often fall short in terms of sensitivity, reproducibility, and performance in complex matrices. By contrast, the surface-grown MOF on the 3D-printed impact zone ensures a more efficient interaction with the analyte, leading to enhanced fluorescence quenching and improved analytical performance. The use of 3D printing enables the fabrication of a custom-designed device that fits directly into standard instrumentation, facilitating F^3^S. These features make 3D printing a superior alternative to conventional materials such as paper-based platforms, offering greater precision, adaptability, and structural robustness.

The successful fabrication of the device was demonstrated through fluorescence, FTIR, SEM, and XRD characterization, which confirms the successful anchoring and growth of the Tb-MOF on the functionalized 3D-printed surface. These results validate the effectiveness of the in situ synthesis strategy, which enables strong integration of the MOF while preserving its optical properties. The developed system was evaluated to extract and sense tetracyclines and achieves a LOD of 1.6 μg L^–1^ in aqueous media (1.8 and 0.5 μg L^–1^ in seawater and milk, respectively), substantially better than the 140 μg L^−1^ obtained using the MOF in dispersive mode and similar to that achieved by HPLC with fluorescence detection. Additionally, the use of the 3D-printed device provides a simpler and more effective approach to minimizing matrix effects in complex samples. This positions the device as a viable alternative to chromatographic techniques, offering comparable sensitivity but at a fraction of the cost and complexity. Furthermore, the analytical figures of merit obtained with this platform, including sensitivity and reproducibility surpass those reported for similar MOF-based systems in the literature. The system shows excellent reproducibility (RSD < 12%) and recovery values above 94% in complex matrices such as seawater and milk, demonstrating both robustness and applicability in real-world scenarios. Additionally, the application of the method to complex matrices demonstrates that potential interferents, such as ions or structurally related compounds, do not significantly affect its performance. Each complete unit can be produced for approximately €1.60 (only €0.5 the modified impact zone), and the device is fully compatible with conventional fluorometers, requiring no hardware modifications. Although the method does not reach the extremely low detection limits obtainable by HPLC–MS, it offers a much more accessible and operationally simpler solution. This makes it particularly attractive for routine monitoring, especially in settings where access to high-end instrumentation is limited. Possible limitations of the proposed device relate primarily to its single-use design, which was chosen to minimize material waste and reduce the consumption of organic eluents. Additionally, the robustness and applicability of the method could be further enhanced by conducting other validation studies. Furthermore, other complex matrix, such as other waters and honey, can be evaluated in the future. However, these investigations fall beyond the scope of the present work, which focuses on introducing a novel 3D-printed platform combined with a MOF for sensing applications.

Overall, this work illustrates the potential of combining 3D printing and MOF materials to create multifunctional, customizable, and high-performance analytical devices. The proposed system not only offers significant improvements over traditional dispersive formats but also matches the performance of more sophisticated techniques like HPLC-FLD, at a lower cost and with simpler operation. It stands as a promising tool for decentralized, sustainable, and efficient monitoring of emerging contaminants such as tetracyclines in seawater and milk.

## Supplementary Information

Below is the link to the electronic supplementary material.

ESM 1 (PDF 545 KB)

## Data Availability

Data is provided within the manuscript or supplementary information files.
