# Supplementary material for: Front-face fluorescence of tetracyclines using a modulable 3D-printed platform modified with an extraction and sensing sorbent based on rare-earth metal–organic frameworks
Source: Mikrochim Acta. 2025 Jun 8;192(7):410. doi: 10.1007/s00604-025-07274-y (PMC12145305; doi:10.1007/s00604-025-07274-y)
Supplement: Supplementary file 1 — (PDF 545 KB) [file 604_2025_7274_MOESM1_ESM.pdf]

SUPPLEMENTARY INFORMATION

Front-face fluorescence of tetracyclines using a modulable 3D-printed platform modified with an extraction and sensing sorbent based on rare-earth metal-organic frameworks

Laura Alcázar-Escobedo, Noelia Campillo-Tamarit, Ernesto Francisco Simó-Alfonso, Enrique Javier Carrasco-Correa*

CLECEM group, Department of Analytical Chemistry, University of Valencia, 46100-Burjassot, Valencia, Spain.

Pages: 5

Figures: 4

Tables: 1

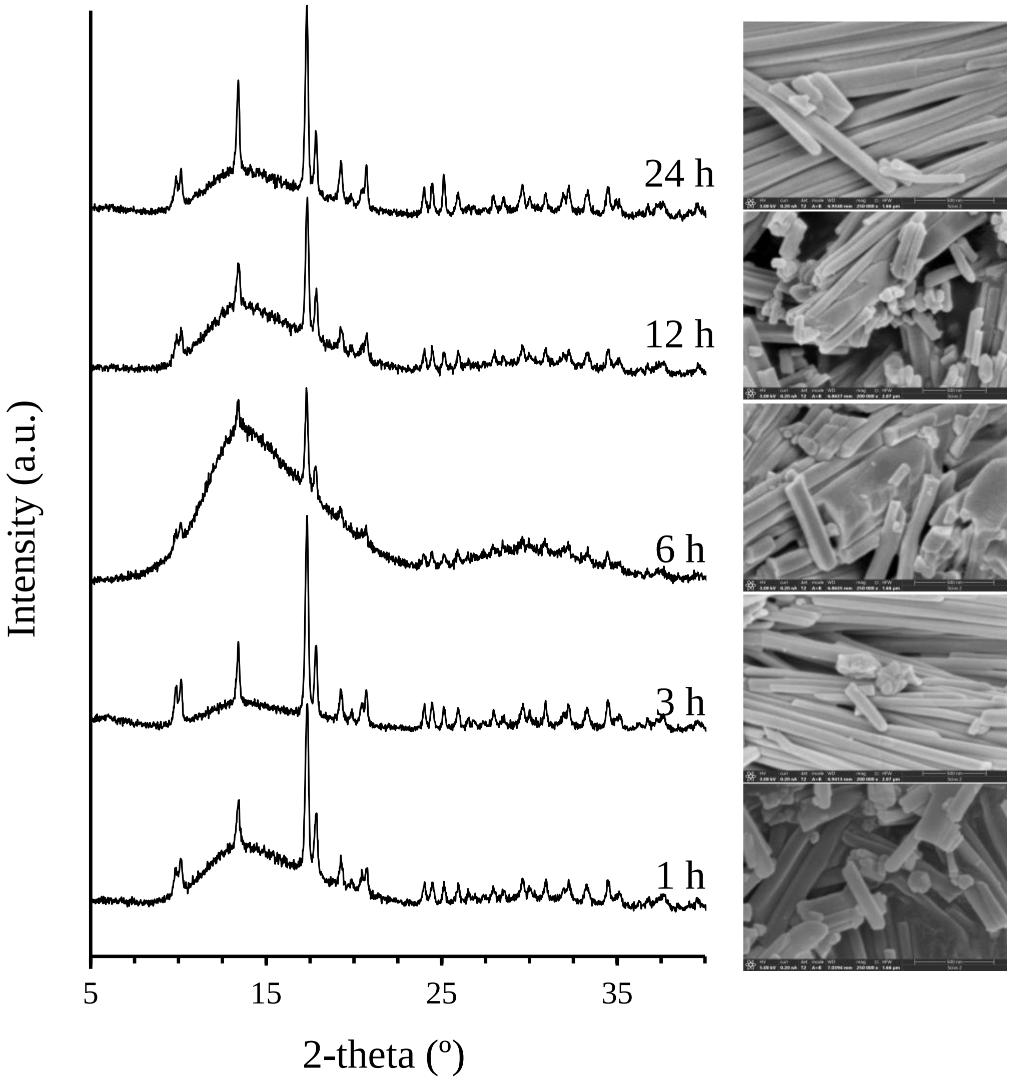

Fig. S1. XRD patterns and SEM micrographs for the $Tb(BTC)(H_2O)_6$ MOF prepared un powder at different synthesis times.

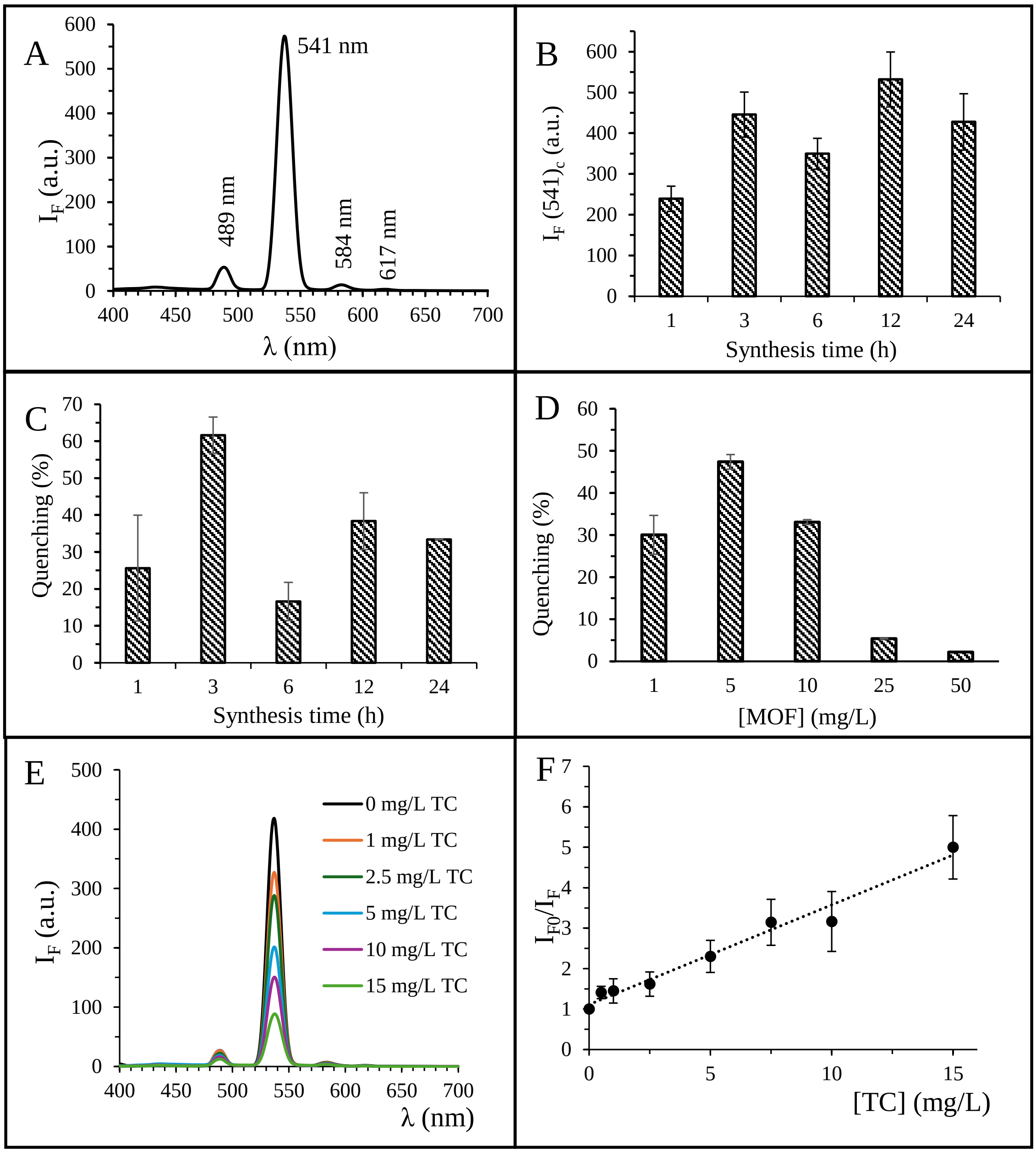

Fig. S2. The fluorescence emission spectrum (λ_{exc} = 260 nm) of the dispersed in water $Tb(BTC)(H_2O)_6$ MOF (A). The fluorescence intensity at 5 mg L^{-1} of MOF (B) and quenching effect at 5 mg L^{-1} of MOF in presence of 5 mg L^{-1} of tetracycline (C) at different reaction times measured at 541 nm. Quenching effect at different $Tb(BTC)(H_2O)_6$ MOF concentrations in presence of 5 mg L^{-1} of tetracycline (D). Calibration curves of tetracycline: spectra (E) and plot (F).

Table S1. Analytical performance of the $Tb(BTC)(H_2O)_6$ MOF dispersed fluorescence, HPLC-FLD and HPLC-MS methods.

Analytical parameter	$Tb(BTC)(H_2O)_6$ MOF dispersed fluorescence method	HPLC-FLD method	HPLC-MS method
LOD	140 µg/L	0.3 µg/L	7 ng/L
RSD (%)	6.6	5.1	6.9
R^2	0.9750	0.9984	0.9978
Range limit	0.4-18 mg/L	0.001-25 mg/L	0.022-1000 µg/L
Sensitivity[a] (L/mg)	0.250	0.099	1.009

[a] Since the $Tb(BTC)(H_2O)_6$ MOF dispersed fluorescence method uses the Stern-Volmer equation that applies a correction, similar corrections were applied to the HPLC-FLD and HPLC-MS method to obtain the same range of sensitivity.

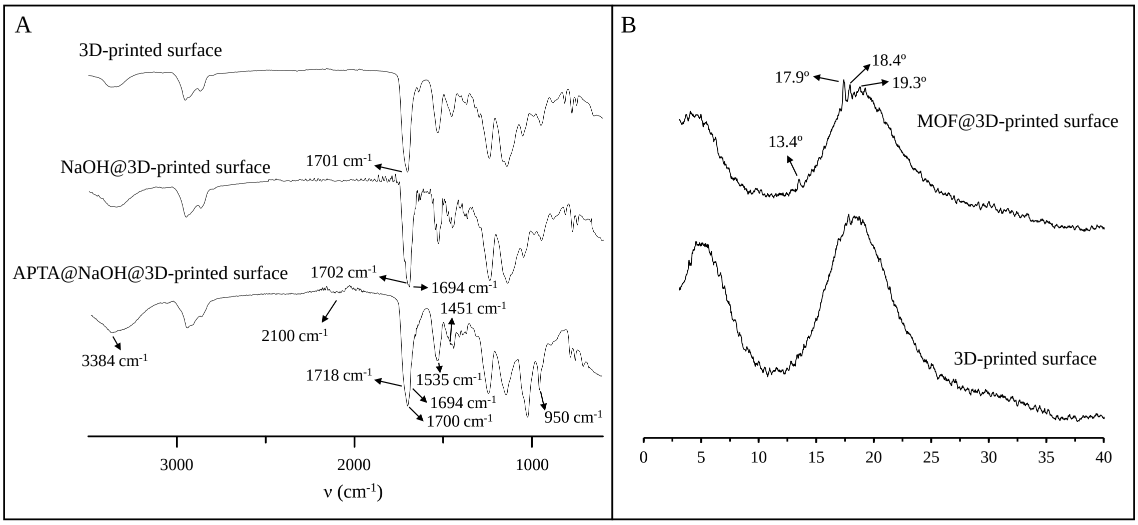

Fig. S3. FTIR spectra of the 3D printed surfaces and the 3D-printed surfaces modified with NaOH and APTA (A) and XRD patterns of the 3D-printed surface and the 3D-printed surface modified with the $Tb(BTC)(H_2O)_6$ MOF (B).

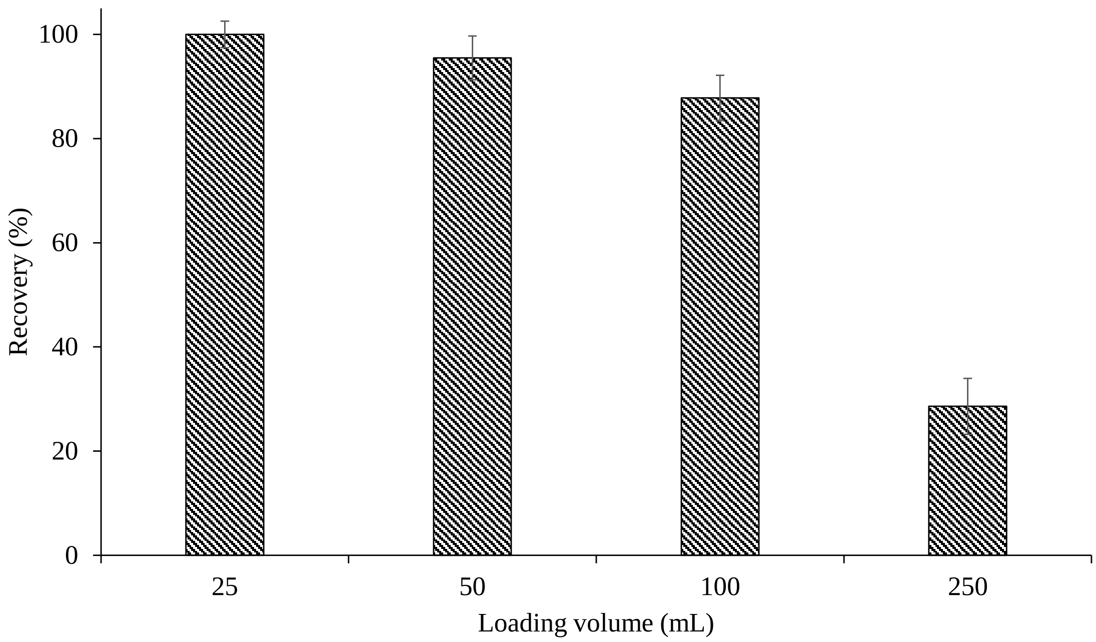

Fig. S4. Recovery obtained for the $Tb(BTC)(H_2O)_6$ MOF growth on the 3D-printed surface to evaluate the break-through volume.
